# Actin Cytoskeleton Remodeling Accompanied by Redistribution of Adhesion Proteins Drives Migration of Cells in Different EMT States

**DOI:** 10.3390/cells13090780

**Published:** 2024-05-02

**Authors:** Alla S. Ilnitskaya, Nikita I. Litovka, Svetlana N. Rubtsova, Irina Y. Zhitnyak, Natalya A. Gloushankova

**Affiliations:** 1 Institute of Carcinogenesis, N.N. Blokhin National Medical Research Center of Oncology, 24 Kashirskoye Shosse, 115478 Moscow, Russia; ilnitskaya.alla@gmail.com (A.S.I.); foxcovert9@gmail.com (N.I.L.); gaart2@gmail.com (S.N.R.); irina.zhitnyak@utoronto.ca (I.Y.Z.); 2 Department of Molecular Genetics, University of Toronto, 661 University Ave, MaRS West, Toronto, ON 5MG 1M1, Canada

**Keywords:** epithelial–mesenchymal transition, cell migration, cell–cell adhesion, adherens junctions, α-catenin, β-catenin, integrin adhesion complexes

## Abstract

Epithelial–mesenchymal transition (EMT) is a process during which epithelial cells lose epithelial characteristics and gain mesenchymal features. Here, we used several cell models to study migratory activity and redistribution of cell–cell adhesion proteins in cells in different EMT states: EGF-induced EMT of epithelial IAR-20 cells; IAR-6-1 cells with a hybrid epithelial–mesenchymal phenotype; and their more mesenchymal derivatives, IAR-6-1-DNE cells lacking adherens junctions. In migrating cells, the cell–cell adhesion protein α-catenin accumulated at the leading edges along with ArpC2/p34 and α-actinin. Suppression of α-catenin shifted cell morphology from fibroblast-like to discoid and attenuated cell migration. Expression of exogenous α-catenin in MDA-MB-468 cells devoid of α-catenin drastically increased their migratory capabilities. The Y654 phosphorylated form of β-catenin was detected at integrin adhesion complexes (IACs). Co-immunoprecipitation studies indicated that α-catenin and pY654-β-catenin were associated with IAC proteins: vinculin, zyxin, and α-actinin. Taken together, these data suggest that in cells undergoing EMT, catenins not participating in assembly of adherens junctions may affect cell migration.

## 1. Introduction

Epithelial–mesenchymal transition (EMT) is a cellular process in the course of which epithelial cells lose epithelial characteristics and gain mesenchymal features. During EMT, epithelial cells lose apico-basal polarity and epithelial integrity and acquire mesenchymal phenotype and migratory activity [1]. EMT contributes to various morphogenetic events in development, is involved in wound healing in adults, and is activated in diseases such as fibrosis or cancer. EMT is essential for initiation of the invasion-metastasis cascade [2]. Recent studies have shown that EMT is also associated with stemness, tumorigenicity, plasticity, and chemotherapy resistance of cancer cells [3,4,5]. It has been realized that in cancer, EMT is not a binary switch between epithelial and mesenchymal states but a spectrum of various intermediate phenotypes, each induced by its own unique combination of mutations and signals from the microenvironment. Cancer cells undergoing EMT exhibit certain mesenchymal traits and express mesenchymal markers but may still retain some epithelial markers, for example, E-cadherin. Numerous studies identified distinct hybrid epithelial/mesenchymal cells in various carcinomas [6].

Normal epithelial cells form typical E-cadherin-catenin transmembrane protein complexes (adherens junctions, AJs) that form a continuous adhesion belt around the apical part of a cell (linear AJs). Linear AJs are associated with the main actin cytoskeletal structure of the epithelial cell, namely, the circumferential actin bundle, and provide stable cell–cell adhesion and epithelial integrity [7,8,9]. Weakening of cell–cell adhesion is the first important step for a cell in moving away from its neighbors during EMT, and disruption of stable AJs may be due to downregulation of E-cadherin or other adhesion protein expression or their post-translational modifications [10]. Dramatic reorganization of the actin cytoskeleton is a major driver of profound changes in cell morphology and cell–cell adhesion during EMT. Recently, we showed that during dynamic EGF-induced EMT, dissolution of circumferential actin bundle and formation of lamellipodia at the cell–cell boundaries accompanied by replacement of stable linear E-cadherin-based AJs with unstable punctate AJs led to disruption of stable cell–cell adhesion [11].

In epithelial cells, α-catenin, which is a part of the cadherin–catenin adhesion complex, is responsible for connecting AJs to filamentous actin (F-actin) that stabilizes their structure [12,13]. α-Catenin consists of three domains: the N-terminal domain, which binds to β-catenin [14,15] and contains an α-actinin-binding site [16], the central domain, which contains a MI vinculin-binding domain (VBD) [17,18,19], and the C-terminal actin-binding domain (ABD) [20]. Single-molecule study demonstrated that mechanical force was required for ABD binding to F-actin [21]. Vinculin additionally recruits actin to cadherin–catenin complexes [9].

Studies of the role of the extrajunctional pool of α-catenin in cells yielded controversial data. In vitro studies showed that α-catenin dimers bound to actin filaments and inhibited Arp2/3-mediated actin polymerization [22]. Earlier, it had been shown that α-catenin–null keratinocytes exhibited an invasive activity in Transwell chambers [23]. In MDCK cells, artificial sequestration of cytosolic α-catenin to mitochondria increased protrusive activity and migration into the wound, whereas sequestration of cytosolic α-catenin to the plasma membrane reduced protrusive activity. shRNA-mediated knockdown of α-catenin did not affect the rate of wound closure in MDCK monolayer [24]. Chemical-induced dimers of α-catenin recruited to membranes induced filopodia extension, though the rate of wound healing was lower than in the control [25]. Thus, it would seem that extrajunctional α-catenin attenuates cell migration. In contrast, knockdown of α-catenin or β-catenin under the control of the neural crest-specific P0 promoter slowed the migration of enteric neural crest cells into embryonic gut. In vitro, these cells and U251 glioblastoma cells with reduced α-catenin or β-catenin expression did not show directional migration [26]. Recently, Mukherjee et al. [27] described α-catenin enrichment at the cell edges at an early stage of fibroblast spreading and its association with integrin adhesion complexes (IACs).

Cell migration requires highly coordinated extension of protrusions at the leading edge, their integrin-mediated adhesion to the extracellular matrix, generation of traction forces, and cell body contraction. Formation of IACs at the leading edge of migrating cells stabilizes lamellipodial protrusions. During lamellipodial extension, nascent adhesions form at the leading edge. In addition to integrin, these structures contain kindlin, talin, focal adhesion kinase (FAK), paxillin, and α-actinin. Some of the nascent adhesions mature into focal complexes, and then into larger focal adhesions that are subsequently disassembled (focal adhesion turnover). IAC maturation involves clustering of integrins, conformational opening of talin and recruitment of vinculin, their cross-linking with actin, and generation of acto-myosin contractility [28]. α-Actinin links integrins to actin and transmits force between integrins and the actin cytoskeleton, which also triggers maturation of IACs [29]. Another component of IACs, zyxin, interacts with the N-terminal actin-binding domain of α-actinin [30]. IACs are important dynamic platforms that recruit Rho- and Rac-specific GEFs and GAPs to control the protrusion–contraction balance in migrating cells [31].

Here, we used several cell models to study the involvement of cell–cell adhesion proteins in the migratory activity of cells in different EMT states. We investigated EGF-driven EMT in IAR-20 epithelial cells and MDA-MD-468 breast cancer cells and analyzed the migration of IAR-6-1 transformed epithelial cells with a hybrid epithelial/mesenchymal phenotype and their more mesenchymal descendants, IAR-6-1-DNE cells, which lost their AJs as a result of stable expression of a dominant-negative mutant of E-cadherin.

## 2. Materials and Methods

### 2.1. Cell Lines, Constructs, and Transfections

The IAR-20 line of immortalized rat liver epithelial cells and the IAR-6-1 line of rat liver epithelial cells transformed with dimethylnitrosamine were established at the International Agency for Research on Cancer [32]. Both cell lines were kindly provided by Prof. R. Montesano. The MDA-MB-468 human breast cancer cell line was obtained from ATCC (HTB-132). The cells were cultured in DMEM supplemented with 10% fetal bovine serum (FBS) and 1% penicillin-streptomycin at 37 °C with 5% CO_2_. IAR-20 cells were treated with EGF (50 ng/mL) for indicated times. MDA-MB-468 cells were treated with EGF (20 ng/mL) for 10 h.

The actin marker F-tractin (ITPKA-9–40) tagged with tdTomato was a gift from M. Schell (Uniformed Services University, Bethesda, MD, USA) [33]. The mCherry-α-catenin construct was kindly provided by Prof. S.M. Troyanovsky (Northwestern University, Evanston, IL, USA). Transfections were carried out using Lipofectamine^®^ LTX and PLUS™ transfection reagents (Invitrogen, ThermoFisher Scientific, Waltham, MA, USA) according to the manufacturer’s protocol. For transient transfections, live cell imaging was performed 48 h post-transfection. For stable transfection, cells expressing F-tractin-tdTomato or mCherry-α-catenin were obtained after 2 weeks of selection with G418 (0.5 mg/mL).

To suppress α- or β-catenin, ON-TARGETplus rat *Ctnna1* siRNA (50 nM) or ON-TARGETplus rat *Ctnnb1* siRNA (50 nM) and a transfection reagent Dharmafect1 (Horizon Discovery, Cambridge, UK) were used. Anti-GFP siRNA was used as a negative control. At 48 h post-si-α-catenin transfection, cells were lysed and examined by Western blotting or seeded onto etched grid coverslips (Bellco Glass, Vineland, NJ, USA) placed into ibidi 2-well chambers (ibidi, Gräfelfing, Germany) for DIC microscopy and subsequent immunofluorescent staining. At 48 h post-si-β-catenin transfection, cells were examined by Western blotting or immunofluorescence analysis.

### 2.2. Antibodies and Reagents

The following primary mouse monoclonal antibodies were used: anti-E-cadherin, clone 36; anti-β-catenin, clone 14; anti-paxillin, clone 349 (BD Transduction Labs, Franklin Lakes, NJ, USA); anti-zyxin, clone 164D4 (Synaptic Systems, Göttingen, Germany); anti-β-actin, clone 4C2 (Merck, Darmstadt, Germany); anti-vinculin, clone hVin1 (Sigma, Merck, Darmstadt, Germany); anti-α-actinin-1, clone BM-75.2 (Sigma, Merck); anti-α-catenin, clone 15D9 (Enzo Life Sciences, Farmingdale, NY, USA); rabbit polyclonal anti-p34-Arc/ARPC2 (Upstate, Merck, Darmstadt, Germany); anti-pY654-β-catenin (Invitrogen, Thermo Fisher Scientific, Waltham, MA, USA). The secondary goat anti-mouse IgG, IgG1, IgG2a, IgG2b or anti-rabbit IgG antibodies conjugated with AlexaFluor488, 594 or 647 (Jackson Immunoresearch, West Grove, PA, USA) were used. AlexaFluor488-conjugated phalloidin (Molecular Probes, Eugene, OR, USA) or TRITC-conjugated phalloidin (Fluka Chemie, Buchs, Switzerland) was added to the secondary antibodies. For Western blotting, goat anti-mouse and anti-rabbit IgG antibodies conjugated to horse radish peroxidase (Jackson Immunoresearch) were used. Other reagents were obtained from Sigma, Merck.

### 2.3. Immunofluorescence

For fluorescent staining, cells were fixed with 3.7% paraformaldehyde followed by permeabilization with 0.25% Triton X-100 or with 1% paraformaldehyde followed by cold methanol. Fixed specimens were incubated for 40 min with primary antibodies and subsequently for 40 min with secondary antibodies. Mounted samples were examined with a Leica TCS SP5 confocal laser scanning microscope (Leica Microsystems, Wetzlar, Germany) equipped with an HDX PL APO 63× objective or with a Nikon Eclipse Ti-E microscope (Nikon Instruments, Amstelveen, Netherlands) equipped with a Plan Fluor 40× objective and ORCA-flash4.0 camera (Hamamatsu Photonics, Hamamatsu City, Shizuoka, Japan) controlled via NIS-Elements AR software (v.3.22, Nikon Instruments).

### 2.4. Live-Cell Imaging, Tracking, and Kymograph Analysis

Cells were seeded into 35 mm glass bottom culture dishes (MatTek Corporation, Ashland, MA, USA) or 2-well chambers (ibidi). DIC images were obtained using a Nikon Eclipse Ti-E microscope (Plan Fluor 20× objective; ORCA-flash4.0 camera by Hamamatsu Photonics; NIS-Elements AR software v. 3.22 by Nikon Instruments). For cell tracking, the position of the cell’s center of mass throughout a time lapse DIC sequence (1 frame/5 min) was detected using Fiji/ImageJ (v. 2.9.0, NIH, Bethesda, MD, USA). Velocity, track length, and distance were then determined using Microsoft Excel 2016 (Microsoft, Redmond, WA, USA). For kymograph analysis, DIC sequences were shot with a Nikon Eclipse Ti-E microscope (Plan Fluor 100× objective) at 1 frame/3 s. Kymographs were produced using Fiji/ImageJ with the Multiple Kymograph plugin. A straight-line selection was drawn perpendicular to the cell edge. Fluorescence intensity, elongation, and circularity were measured using Fiji/ImageJ. Directionality ratio and mean square displacement were determined using the DiPer macro described in [34]. Live cell imaging of cells expressing F-tractin-tdTomato was performed using Leica TCS SP5 confocal laser scanning microscope (Leica Microsystems) equipped with an HDX PL APO 63× objective. Supplementary Video Files were obtained by generating an uncompressed video from a TIFF image sequence using Fiji/ImageJ and further compressing it using an MP4-to-AVI converter v. 1.17 (Pazera Software, Sosnowiec, Poland).

### 2.5. Co-Immunoprecipitation and Western Blotting

Cells growing on 10 cm Petri dishes were washed twice with ice-cold wash buffer (10 mM Tris-HCL, pH 7.5, 0.5 mM EDTA, 150 mM NaCl), scraped into 200 µL of lysis buffer per dish (wash buffer with 0.5% Na deoxycholate, 1% NP-40) supplemented with protease inhibitor cocktail (Roche, Merck, Darmstadt, Germany) and phosphatase inhibitor cocktail diluted 1:100 (Sigma, Merck), and incubated at +4 °C with rotation for 20 min followed by centrifugation at 17,000× *g*. A total of 100 µL of the lysate was used for the input/positive control. The remaining volumes of the lysates were incubated with primary antibodies at +4 °C with rotation for 20 min. Then, Protein A or G Agarose beads (Invitrogen, Thermo Fisher Scientific) were added (50 µL per tube; prewashed with wash buffer and blocked with 1% BSA overnight), and the suspensions were incubated at +4 °C with rotation for 2 h. After the incubation, the lysates were centrifuged for 1 min at 900× *g*. Pellets were quickly washed 3 times with ice-cold wash buffer using centrifugation at 2500× *g*, resuspended in sample buffer, and heated to +95 °C for 10 min, then lysis buffer was added to the heated pellets. Western blot analysis was conducted according to the standard protocol, using PAAG electrophoresis, transfer onto 0.45 µm nitrocellulose membrane Hybond-C (RPN.303C), and detection using Pierce ECL Western Blotting Substrate (Thermo Fisher Scientific). Chemiluminescence images were captured by Image Quant LAS4000 (GE Healthcare, Freiburg, Germany).

## 3. Results

### 3.1. EGF-Induced EMT of IAR-20 Epithelial Cells

**Figure 1:**
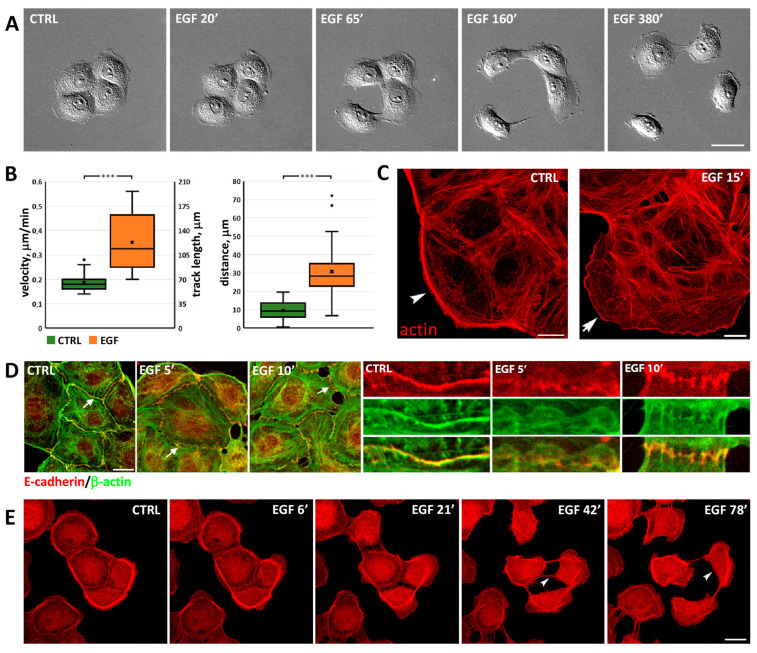
Changes in morphology, actin cytoskeleton, and AJs in IAR-20 epithelial cells during EGF-induced EMT. (**A**) Scattering of IAR-20 cells. Selected frames from Supplementary Video S1. Cells disrupt cell–cell contacts and migrate over the substrate. Scale 20 µm. (**B**) Migratory characteristics of control vs. EGF-treated IAR-20 cells (6 h, N = 23 cells, representative of 3 independent experiments). ***—*p* < 0.001. (**C**–**E**) Actin cytoskeleton remodeling in IAR-20 cells undergoing EMT. (**C**) Control cells display marginal actin bundles (arrowhead). EGF induces protrusive activity at the free cell edges: lamellipodia containing actin network begin to form at the leading edge (arrow). Scale 10 μm. (**D**) Dissolution of the circumferential actin bundle and replacement of linear E-cadherin-based AJs by radial (punctate) AJs during early EMT. Arrows indicate the cell–cell boundaries shown at higher magnification on the right. Scale 10 μm. (**E**) IAR-20 cells stably expressing F-tractin-tdTomato undergo EMT. Arrowheads indicate contraction of a cell’s rear. Selected frames from Supplementary Video S2. Scale 20 μm.

The IAR-20 line of immortalized epithelial cells was established from rat liver [32]. Single IAR-20 cells were discoid; in sparse culture, cells formed islands with stable cell–cell adhesions; in dense culture, these islands merged into a monolayer. We found that in IAR-20 cells, epidermal growth factor (EGF) promoted dynamic EMT (Figure 1A,B; Supplementary Video S1). Within 5–10 min after the addition to culture medium, EGF induced protrusive activity both at the free cell edges and at the cell–cell boundaries. Live cell imaging showed that cells began to migrate, disrupting existing cell–cell contacts. Migrating cells could establish cell–cell contacts with neighboring cells, but unlike stable adhesions between control IAR-20 cells, these were transient and could quickly disassemble.

In IAR-20 cells treated with EGF, changes in cell morphology corresponded with reorganization of the actin cytoskeleton. As was revealed by immunofluorescence microscopy, control IAR-20 cells possessed marginal actin bundles at the free edges, circumferential actin bundles co-localized with linear AJs, and randomly oriented straight actin bundles in the cytoplasm. Treatment of IAR-20 cells with EGF resulted in dramatic changes in the actin cytoskeleton. Already after 5–15 min treatment with EGF, marginal actin bundles fragmented or disappeared, and lamellipodia filled with the actin filament network extended at the free edges (Figure 1C). Circumferential actin bundles at the cell–cell boundaries disappeared, and lamellipodia filled with the actin filaments formed instead. In IAR-20 cells undergoing EMT, reorganization of the actin cytoskeleton was accompanied by fast dissolution of linear AJs and, beginning from 10–15 min after the addition of EGF, replacement of linear AJs with punctate AJs connected with straight (radial) actin bundles that were oriented perpendicular to the intercellular boundary. Immunostaining showed that in IAR-20 cells treated with EGF punctate, AJs were formed by E-cadherin (Figure 1D).

To characterize the dynamics of actin cytoskeleton remodeling during EMT in live cells, we established a line of IAR-20 cells stably expressing the F-actin marker F-tractin-tdTomato [33]. Using confocal microscopy, we observed activation of actin network polymerization at the free cell edges, disassembly or fragmentation of marginal actin bundles, and dissolution of circumferential actin bundles within 5–15 min after addition of EGF (Figure 1E and Supplementary Video S2). Polymerization of the actin network in lamellipodia at the leading edges and contractility of the rear promoted front–rear polarization and cell migration.

### 3.2. Migratory Activity of IAR-6-1 Cells Exhibiting Hybrid Epithelial–Mesenchymal Phenotype and Their Descendant IAR-6-1-DNE Cells

**Figure 2:**
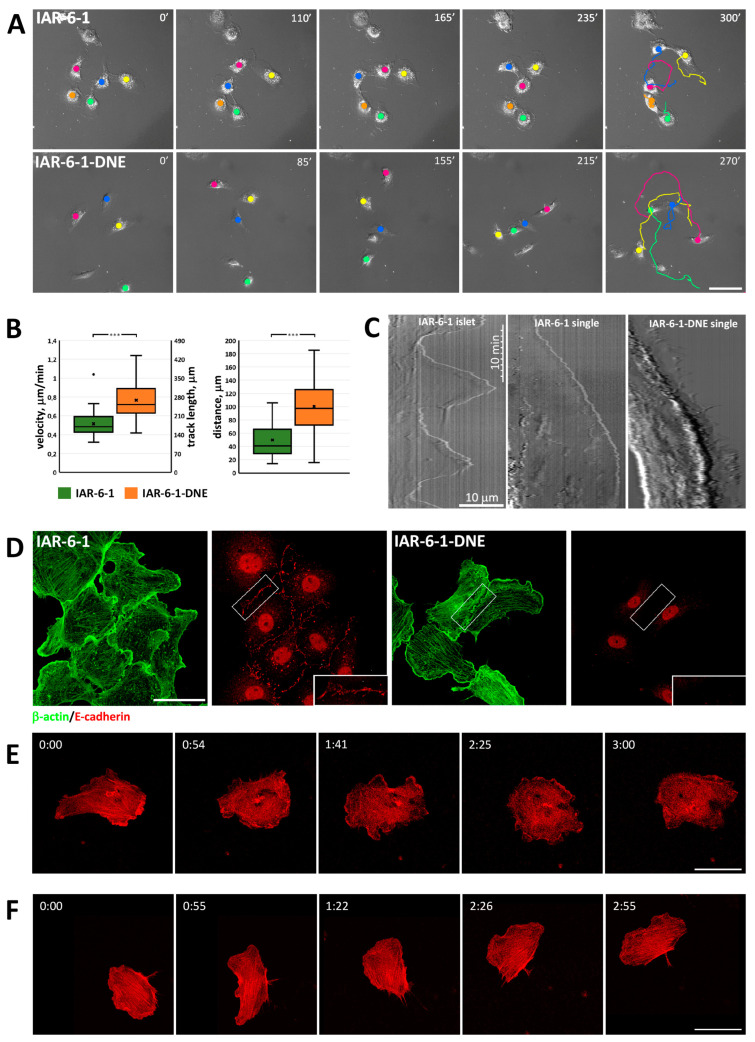
Differences in morphology and migratory characteristics of IAR-6-1 cells with hybrid epithelial–mesenchymal phenotype and IAR-6-1-DNE cells with mesenchymal phenotype. (**A**) Migration of IAR-6-1 cells and IAR-6-1-DNE cells over substrate. Selected frames from Supplementary Videos S3 and S4. IAR-6-1 cells retain the ability to establish cell–cell contacts (Supplementary Video S3), while IAR-6-1-DNE cells do not form even transient cell–cell contacts (Supplementary Video S4). Scale 50 μm. (**B**) Migratory characteristics of IAR-6-1 cells and IAR-6-1-DNE cells (N = 25 cells, representative of 3 independent experiments). ***—*p* < 0.001. (**C**) Representative kymographs of an IAR-6-1 cell joined by AJs with a neighboring cell in an islet, a single migrating IAR-6-1 cell, and a migrating IAR-6-1-DNE cell. (**D**) Fluorescent staining for the actin cytoskeleton and AJs. Boxed regions are shown in higher magnification. IAR-6-1 cells form punctate E-cadherin-based AJs. IAR-6-1-DNE cells lost the ability to assemble AJs. Scale 25 μm. (**E**) An IAR-6-1 cell transiently transfected with F-tractin-tdTomato in contact with other cells (Supplementary Figure S2). Selected frames from Supplementary Video S5. Numerous pseudopodia are formed at the free edges; however, the cell does not migrate. Scale 50 μm. (**F**) A migrating single IAR-6-1-DNE cell transiently transfected with F-tractin-tdTomato. Selected frames from Supplementary Video S6. Scale 50 μm.

As another model of EMT states, we used the IAR-6-1 line that had been established from the IAR-6 line of rat liver epithelial cells transformed in vitro with dimethylnitrosamine [32]. As a result of neoplastic transformation, these cells underwent partial EMT and acquired migratory activity. Western blot analysis and immunofluorescent staining showed that transformed IAR-6-1 epithelial cells maintained E-cadherin expression; therefore, IAR-6-1 cells possessed a hybrid epithelial–mesenchymal phenotype [35]. IAR-6-1 cells were polygonal or discoid and randomly migrated over underlying surface, both individually and collectively (Figure 2A, Supplementary Video S3). When a single migrating cell collided with another, they could form cell–cell contacts and transiently cease to migrate; however, unlike in normal epithelial cells, cell–cell contacts between IAR-6-1 cells were unstable and could break. Upon breaking of the cell–cell contact, the cells would resume their migration.

We also used IAR-6-1 cells stably expressing a dominant-negative mutant form of E-cadherin with a W156A mutation within the extracellular N-terminal domain (IAR-6-1-DNE; [36]), which completely abolished AJ formation [37]. IAR-6-1-DNE cells possessed a fibroblast-like phenotype with frontal lamellipodia and a contracting rear or a keratocyte-like phenotype with a wide flat leading lamellipodium. IAR-6-1-DNE cells never formed islands and, unlike the parental IAR-6-1 cells, exhibited only individual migration on a 2D substrate (Figure 2A, Supplementary Video S4). If a migrating IAR-6-1-DNE cell collided with another cell, they failed to establish cell–cell contact and migrated away from each other. Analyzing DIC live cell videos, we compared migratory characteristics of IAR-6-1 and IAR-6-1-DNE cells and found that mean cell velocity, track length, and distance between the start and end points were significantly higher for IAR-6-1-DNE cells in comparison with IAR-6-1 cells (Figure 2B). Kymographs of the active edge of IAR-6-1 cells in contact with other cells were very different from kymographs of single migrating IAR-6-1 cells and IAR-6-1 DNE cells (Figure 2C). Contacting IAR-6-1 cells slowly extended and retracted flat lamellipodia on the free edges without any discernible forward movement. In contrast, we observed increased lamellipodia dynamics in individually migrating IAR-6-1 cells and especially in IAR-6-1 DNE cells, where the overall continual advancement of a leading edge was accompanied by numerous rapid and short protrusions and retractions.

The actin cytoskeleton in transformed IAR-6-1 cells with a hybrid epithelial–mesenchymal phenotype was different from the actin cytoskeleton in normal epithelial cells. IAR-6-1 cells did not have marginal actin bundles but instead formed numerous lamellipodia filled with actin filament network with maximum brightness of actin staining detected at the front of lamellipodia (Figure 2D,E). In the cytoplasm, randomly oriented straight actin bundles were observed. IAR-6-1 cells formed punctate E-cadherin-based AJs (puncta or streaks) that were associated with short straight actin bundles. IAR-6-1-DNE cells also formed lamellipodia filled with a network of actin filaments (Figure 2D,F). The thin stress fibers of IAR-6-1-DNE cells exhibiting keratocyte-like crawling locomotion were aligned perpendicular to the direction of migration. In IAR-6-1-DNE cells with the fibroblast-like phenotype, most stress fibers were oriented perpendicular to the leading edge. Unlike IAR-6-1 cells, IAR-6-1-DNE cells did not form AJs, although these cells could assemble cadherin–catenin complexes, as was shown by co-immunoprecipitation (Supplementary Figure S1). Presumably, mutant E-cadherin abolished cis-interactions of E-cadherin molecules and formation of adhesion plaques on the cell membrane, as described by Troyanovsky [38].

### 3.3. Redistribution of α-Catenin in Cells in Different EMT States

**Figure 3:**
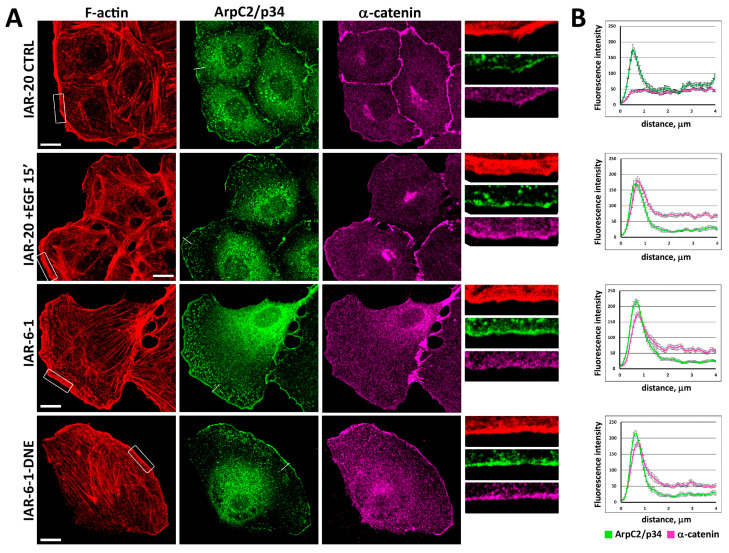
Distribution of F-actin, ArpC2/p34, and α-catenin in cells in different EMT states. (**A**) Immunofluorescent staining. In control and EGF-treated IAR-20 cells and in IAR-6-1 cells, α-catenin accumulated in AJs. In IAR-20 cells undergoing EGF-induced EMT, IAR-6-1, and IAR-6-1-DNE cells, immunostaining shows enrichment of α-catenin at the leading edge in the zone of actin polymerization. Boxed regions are shown at higher magnification on the right. Scale 10 μm. (**B**) Fluorescence intensity along straight lines (N = 25 from 10–12 cells from 2 independent experiments). Examples of lines are shown in the green channel.

Early stages of EMT include reorganization or complete disappearance of E-cadherin-based AJs. Using confocal microscopy, we studied the distribution of α-catenin in the cells undergoing EMT induced by EGF and cells in different EMT states. In control IAR-20 and IAR-6-1 cells contacting with other cells, α-catenin accumulated in the zones of cell–cell interaction participating in the assembly of AJs (Figure 3). In IAR-20 cells treated with EGF, migrating IAR-6-1 cells, and IAR-6-1-DNE cells, we observed the enrichment of α-catenin in the zones of actin network assembly at the leading edge. At the leading edges of migrating cells, α-catenin was detected in the zone of accumulation of actin-nucleating Arp2/3 complex, which was visualized by staining for the ArpC2/p34 subunit of the complex.

**Figure 4:**
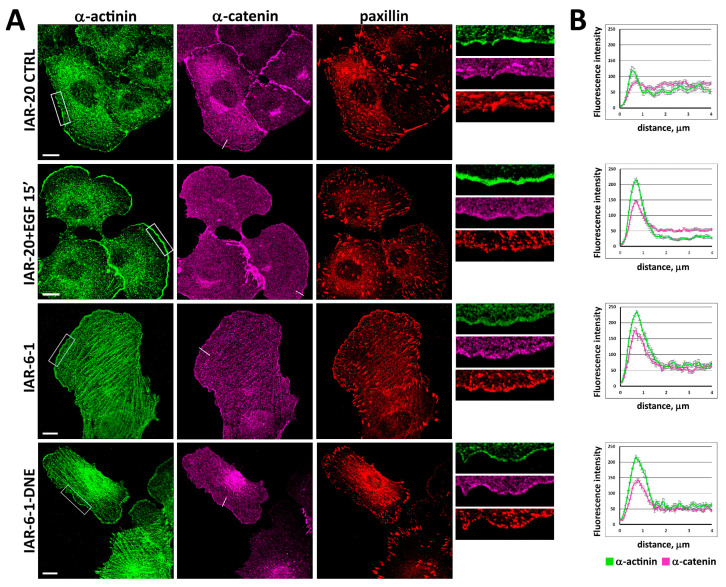
Enrichment of α-actinin and α-catenin and formation of integrin adhesion complexes at the leading edge of migrating cells (EGF-treated IAR-20, IAR-6-1, IAR-6-1-DNE). (**A**) Immunofluorescent staining. Boxed regions are shown at higher magnification on the right. Scale 10 μm. (**B**) Fluorescence intensity along straight lines (N = 25 from 10–12 cells from 2 independent experiments). Examples of lines are shown in the magenta channel.

In EGF-treated IAR-20 cells, migrating IAR-6-1 cells, and IAR-6-1-DNE cells, we also detected the enrichment of α-actinin at the leading edge and its co-localization with α-catenin (Figure 4). Immunofluorescence staining of paxillin demonstrated that numerous IACs formed at the leading edge region that was enriched with α-catenin and α-actinin.

### 3.4. α-Catenin Is Involved in Cell Migration

**Figure 5:**
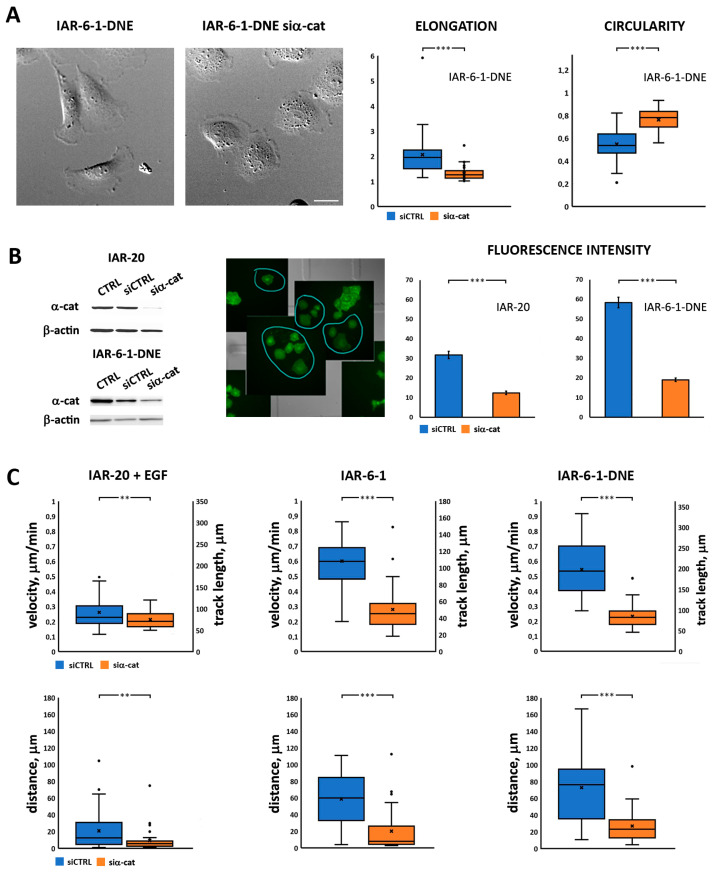
α-Catenin siRNA knockdown alters morphology and attenuates migration of cells in different EMT states. (**A**) Left—effect of α-catenin suppression on morphology of IAR-6-1-DNE cells. Right—elongation and circularity indexes for control IAR-6-1-DNE cells vs. IAR-6-1-DNE cells with suppressed α-catenin (N = 30 cells, representative of 3 independent experiments). Scale 10 μm. (**B**) Left—Western blotting of cells at 48 h after α-catenin siRNA transfection. Center—a representative example of immunofluorescence staining for α-catenin superimposed on the last frame of the DIC image sequence, IAR-20 cells. Blue circles indicate cells with decreased α-catenin expression. Right—mean fluorescent intensity of control and α-catenin-suppressed cells (N = 30 cells, representative of 3 independent experiments). (**C**) Velocities, track lengths, and distances of single cells for EGF-treated IAR-20 (6 h), IAR-6-1 cells (3 h), and IAR-6-1-DNE cells (6 h) (N = 30 cells, representative of 3 independent experiments). **—*p* < 0.05, ***—*p* < 0.001.

To examine whether α-catenin is involved in the migration of cells undergoing EMT, we analyzed the behavior of cells with suppressed α-catenin expression (Figure 5A). Western blot analysis showed that treatment with α-catenin siRNA successfully depleted α-catenin levels (Figure 5B). A decrease in α-catenin expression resulted in dramatic cell shape changes, particularly evident in IAR-6-1-DNE cells. In contrast with polarized control IAR-6-1-DNE cells, many IAR-6-1-DNE cells at 24–72 h post-siRNA transfection were discoid-shaped (Figure 5A). Correspondingly, in the IAR-6-1-DNE cell population after the suppression of α-catenin, a significant decrease in mean cell elongation and an increase in circularity were observed. At 48 h post-siRNA transfection, we studied the migratory activity of the cells seeded on etched grid coverslips using DIC live cell imaging with subsequent immunofluorescent staining for α-catenin (Figure 5B). For individually migrating cells displaying low intensity of α-catenin staining, velocity, track length, and the distance between the start and end points were determined (Figure 5C). We found a statistically significant reduction in velocity, track length, and distance in α-catenin-suppressed cells in comparison with control for EGF-treated IAR-20, IAR-6-1, and IAR-6-1-DNE cultures. Compared to control cells, when α-catenin was suppressed in IAR-20 cells, a decrease in their migration velocity was observed (0.26 ± 0.02 μm/min vs. 0.21 ± 0.01 μm/min, *p* < 0.05; data are presented everywhere as mean ± SEM), in the length of the track traveled in 6 h (94 ± 7 µm vs. 77 ± 2 µm, *p* < 0.05), and in the distance between the initial and end positions of the cells (21 ± 4 µm vs. 9 ± 3 µm, *p* < 0.05). IAR-6-1 cells were observed for 3 h instead of 6 h, because after 3 h of observation, the majority of selected cells joined into islands and ceased individual migration. Suppression of α-catenin led to a decrease in migration speed (0.60 ± 0.04 vs. 0.28 ± 0.03 μm/min, *p* < 0.001), track length (102 ± 5 vs. 50 ± 5 μm, *p* < 0.001), and the distance between the initial and final positions of the cells (59 ± 6 vs. 20 ± 4 μm, *p* < 0.001). The suppression of α-catenin in IAR-6-1-DNE cells dramatically reduced their migration velocity (0.54 ± 0.03 μm/min in control cells and 0.23 ± 0.01 μm/min in α-catenin siRNA treated cells) (*p* < 0.001), the track traveled in 6 h (194 ± 12 µm vs. 83 ± 5 µm, *p* < 0.001), and the distance between the initial and final positions of the cells (73 ± 7 µm vs. 27 ± 3 µm, *p* < 0.001).

**Figure 6:**
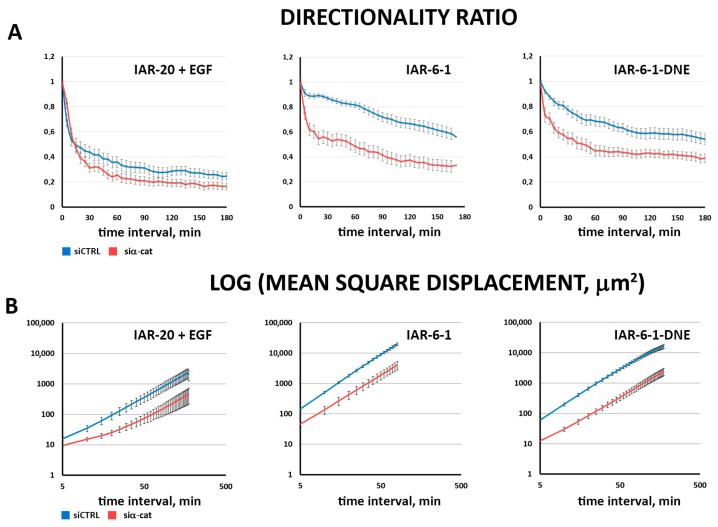
Suppression of α-catenin leads to a decrease in migration velocity and directionality. (**A**) Directionality ratio analysis. (**B**) Mean square displacement analysis. (N = 30 cells, representative of 3 independent experiments; data are presented as mean ± SEM).

For these cells, we also measured two more migration parameters, directionality ratio (DR) and mean square displacement (MSD) (Figure 6; [34]). While DR directly reflects the persistence of cell migration, MSD is a more complex characteristic, depending on both cell velocity and persistence. Both these indices are in accordance with previous calculations (Figure 5C), showing decreased velocity and directionality in cells with suppressed α-catenin expression.

**Figure 7:**
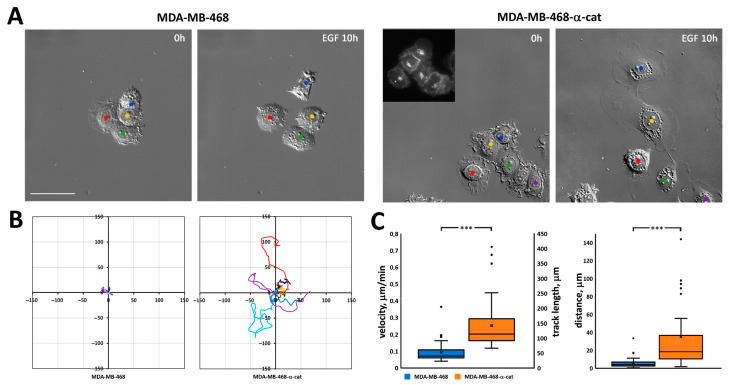
(**A**) EGF-induced EMT in MDA-MB-468 cells. Left—control MDA-MB-468 cells lacking α-catenin; right—MDA-MB-468 cells expressing exogenous mCherry-α-catenin. Insert—expression of mCherry-α-catenin in MDA-MB-468-α-catenin cells. First and last frames from Supplementary Videos S7 and S8. Scale 50 μm. (**B**) Tracks of migrating MDA-MB-468 and MDA-MB-468-α-catenin cells during 10 h of EGF-induced EMT (N = 10 cells, representative of 2 independent experiments). (**C**) Track length, velocity, and distance of migrating MDA-MB-468 and MDA-MB-468-α-catenin cells during 10 h of EGF-induced EMT (N = 35 cells, representative of 2 independent experiments). ***—*p* < 0.001.

To directly examine the involvement of α-catenin in cell migration, we used MDA-MB-468 human breast cancer cells, originally devoid of α-catenin expression [39]. MDA-MB-468 cells were poorly spread and hardly migrated in the presence of EGF (Figure 7A, left, Figure 7B, left; Supplementary Video S7). Following transfection of full-length α-catenin, their migratory behavior dramatically changed; MDA-MB-468 cells expressing exogenous α-catenin were slightly better spread initially and responded well to EGF stimulation, and many cells formed large leading lamella and migrated away from the starting point (Figure 7A, right, Figure 7B, right; Supplementary Video S8). Analysis of migration characteristics confirmed our observations; track length, velocity, and distance between the start and end points were all significantly greater for MDA-MB-468 cells transfected with α-catenin (track length 182 ± 13 μm vs. 52 ± 6 μm for parental MDA-MB-468 cells, *p* < 0.001; velocity 0.30 ± 0.02 μm/min vs. 0.09 ± 0.01 μm/min, *p* < 0.001, and distance 46 ± 6 vs. 6 ± 1 μm, *p* < 0.001; Figure 7C).

### 3.5. pY654-β-Catenin Enrichment at Integrin Adhesion Complexes

**Figure 8:**
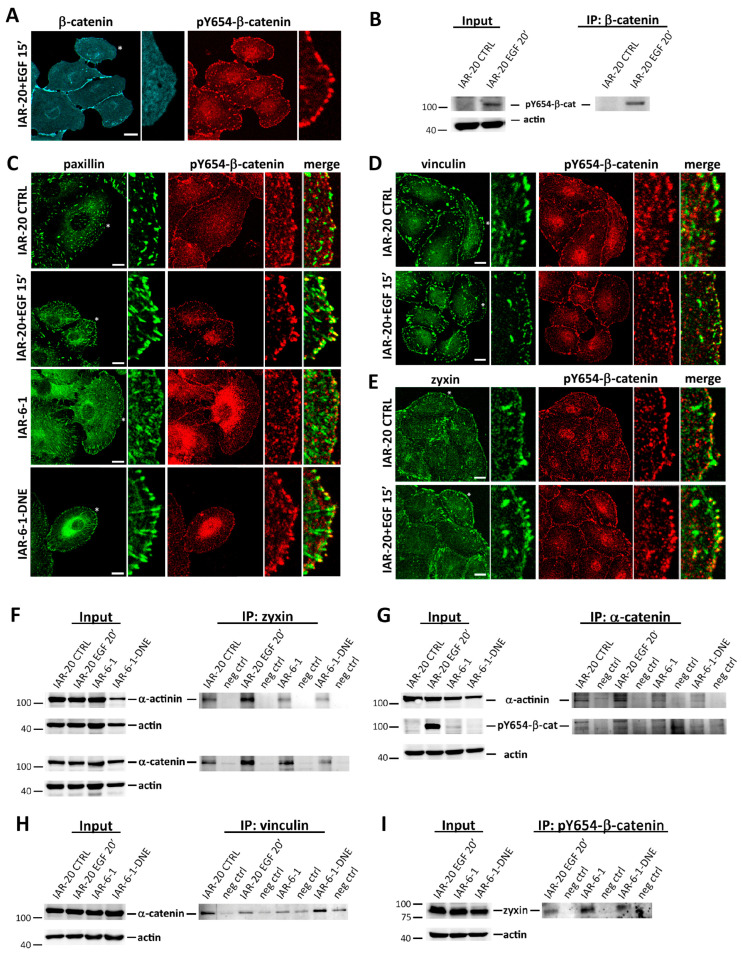
Y654-phosphorylated β-catenin associates with IACs. (**A**) Fluorescent staining of EGF-treated IAR-20 cells for β-catenin and pY654-β-catenin. pY654-β-catenin localizes to AJs and newly formed IACs. Region denoted by an asterisk is shown at higher magnification on the right. Scale 10 μm. (**B**) In IAR-20 cells during EMT, increased protein level of pY654-β-catenin was observed. Total cell lysates of sparse cultures of IAR-20 cells and IAR-20 cells treated with EGF for 20 min were immunoprecipitated for β-catenin and probed with antibodies for pY654-β-catenin. (**C**–**E**) Co-localization of pY654-β-catenin with paxillin (**C**), vinculin (**D**), and zyxin (**E**) at the leading edges of EGF-treated IAR-20, IAR-6-1, and IAR-6-1-DNE cells. Regions denoted by asterisks are shown at higher magnification on the right. Scale 10 μm. (**F**–**I**) pY654-β-catenin and α-catenin interact with IAC proteins—zyxin, vinculin, and α-actinin. Total cell lysates of sparse cultures of control IAR-20 cells, IAR-20 cells treated with EGF for 20 min, IAR-6-1, and IAR-6-1-DNE cells were immunoprecipitated for zyxin (**F**), α-catenin (**G**), vinculin (**H**), or pY654-β-catenin (**I**) and probed with the indicated antibodies.

Previous studies suggested that activated tyrosine kinases phosphorylate β-catenin at Y654, which leads to the dissociation of β-catenin from E-cadherin [40]. We analyzed if these phosphorylation events occur in IAR-20 cells stimulated with EGF in migrating IAR-6-1 and IAR-6-1-DNE cells (Figure 8A–E). In IAR-20 and IAR-6-1 cells, pY654-β-catenin was detected in AJs. Surprisingly, in addition to the presence of pY654-β-catenin in AJs, we observed the enrichment of pY654-β-catenin at IACs. Using confocal microscopy, we detected co-localization of pY654-β-catenin with paxillin, vinculin, and zyxin in newly formed IACs at the leading edges. In more proximal zones, lamella staining of pY654-β-catenin at IACs was less pronounced. In addition, co-immunoprecipitation (co-IP) analyses showed that α-catenin and the pY654 form of β-catenin interact with IAC proteins: vinculin, α-actinin, and zyxin (Figure 8F–I).

To test the specificity of pY654-β-catenin antibodies, immunofluorescence analysis was performed 48 h after siRNA-mediated depletion of β-catenin in IAR-20 cells. After treatment with EGF for 20 min, immunofluorescent staining for β-catenin and pY654-β-catenin and Western blot analysis for β-catenin were performed. A drastic reduction in pY654-β-catenin fluorescence intensity in the puncta at the leading edge was observed in cells with diminished staining for β-catenin (Supplementary Figure S3), confirming target specificity of the pY654-β-catenin antibodies.

## 4. Discussion

Recent studies demonstrated that EMT may not be a binary switch between fully epithelial and fully mesenchymal states, but instead, a process gradually progressing through different intermediate states, for example, in cancer [6]. In this study, we made use of cell models which represent various EMT states to study the important characteristics of the cells that acquire mesenchymal phenotype—migratory activity. We showed that IAR-20 epithelial cells undergo very fast EGF-driven EMT when, within 10–15 min after the addition of EGF, the activation of actin network polymerization at the free cell edges leading to prominent extension of protrusions and their subsequent attachment to substrate promoted cell migration. Similar but slower HGF-stimulated EMT had been described in MDCK cells, where increased protrusive activity at the free cell edges, attachment of protrusions, and integrin-dependent actomyosin contractility resulted in cell scattering [41]. On the contrary, cytoskeleton remodeling in TGF-β-induced EMT of NMuMG mouse mammary epithelial cells manifested as a slow (24–48 h) assembly of thick stress fibers parallel to stable cell edges; however, this EMT variant was accompanied by a decrease in the number of membrane protrusions [42,43].

In this study, we also compared the migratory behavior of IAR-6-1 transformed cells with a hybrid epithelial–mesenchymal phenotype and punctate unstable E-cadherin AJs [35] and their descendants, IAR-6-1-DNE cells. IAR-6-1-DNE cells stably expressed a dominant-negative mutant form of E-cadherin with a W156A mutation within the extracellular N-terminal domain that completely abolished AJ formation [36]. Unlike IAR-6-1 cells, which could migrate both individually and collectively, IAR-6-1-DNE cells never formed islands and exhibited only individual migration. We found that the loss of the ability to assemble cell–cell adhesive structures affected cell migration: the mean cell velocity, track length, and distance between the start and end points were significantly greater for IAR-6-1-DNE cells in comparison with IAR-6-1 cells.

Weakening of cell–cell adhesion is an important early stage of EMT that precedes cell migration. During EMT, degradation of E-cadherin-mediated AJs may be a consequence of the down-regulation of the expression of E-cadherin or other adhesion proteins or post-translational modifications of E-cadherin, leading to its internalization and degradation [44]. It has also been documented that growth factors, e.g., EGF and HGF, induced tyrosine phosphorylation of β-catenin in several tumor cell lines [45,46]. Tyrosine phosphorylation of β-catenin can affect both its adhesive and signaling functions [47,48]. Y654 phosphorylation by oncogenic tyrosine kinases c-src and Bcr-Abl decreased its binding to E-cadherin [40,49]. Using conditional mouse mutants expressing a phospho-mimicking form of β-catenin (Y654E-β-catenin), it was demonstrated that phosphorylation of β-catenin at Y654 reduced its affinity for cadherins, facilitated additional phosphorylation by PKA at S675, and activated Wnt/β-catenin signaling [50]. It was also shown that phosphorylation of β-catenin by PKA increased β-catenin-driven transcriptional activity of TCF transcription factors [51]. Earlier, using dense cultures of IAR-20 epithelial cells, we revealed that destabilization of cell–cell adhesion at the early stages of EGF-induced dynamic EMT may be driven by actin remodeling at cell–cell boundaries: dissolution of circumferential actin bundles following phosphorylation and degradation of EPLIN, activation of actin network polymerization and lamellipodia formation at cell–cell boundaries, accompanied by degradation of linear AJs and their replacement by dynamic punctate (radial) AJs [11].

In epithelial cells, E-cadherin and catenins act as components of cell–cell adhesion complexes, and their importance in AJ assembly has been extensively documented. Newly synthesized E-cadherin associated with β-catenin translocates to the plasma membrane, even in single cells [52]. E-cadherin association prevents β-catenin from interacting with the destruction complex (APC, axin, glycogen synthase kinase 3β, and casein kinase I) by competing with APC [47]. At a time coincident with the arrival of the E-cadherin/β-catenin complex at the plasma membrane, α-catenin gets incorporated into it [52]. The formation of these complexes does not require cell–cell adhesion; our co-immunoprecipitation experiments demonstrated that IAR-6-1-DNE cells that did not form AJs due to W156A mutation of E-cadherin could still assemble cadherin–catenin complexes.

There has been a small number of conflicting studies on the activating vs. inhibiting role of extrajunctional α-catenin in the regulation of actin dynamics [22,23,24,25,26,27]. In this study, we detected the enrichment of α-catenin at the leading edge of migrating cells in various EMT states and its association with α-actinin. It was typical for IAR-20 cells stimulated with EGF, single migrating IAR-6-1 cells, and IAR-6-1-DNE cells. We hypothesize that the adhesion protein α-catenin that is not participating in the formation of AJs may be involved in the activation of cell migration. We observed that the introduction of α-catenin siRNA into IAR-20, IAR-6-1, and IAR-6-1-DNE cells led to changes in cell morphology. It was especially pronounced in IAR-6-1-DNE cells, where cells with decreased α-catenin level were discoid or polygonal, while control IAR-6-1-DNE cells had polarized fibroblast-like morphology. Migration velocity and persistence of cells with reduced α-catenin expression were decreased for all three cell lines.

We also introduced mCherry-α-catenin into α-catenin-deficient MDA-MB-468 cells and compared the effect of EGF on control and α-catenin-expressing cells. Treatment with EGF reduced cell–cell adhesion in control MDA-MB-468 cells; however, the majority of the cells did not migrate away from the neighboring cells. In contrast, in MDA-MB-468 cells transfected with mCherry-α-catenin, we observed many migrating cells with wide leading lamella during treatment with EGF. We found statistically significant increases in velocity, track length, and distance in EGF-treated cells expressing mCherry-α-catenin in comparison with cells lacking α-catenin. Thus, our data indicate that during EMT, when cell–cell adhesion is weakened, extrajuncional α-catenin can accumulate at the active edge, stimulate the transition from immobile epithelial cells to migrating mesenchymal-like cells.

Surprisingly, in migrating IAR-20, IAR-6-1, and IAR-6-1-DNE cells, we observed redistribution of α-catenin’s binding partner, β-catenin, and its accumulation at IACs, predominantly at the active cell edges. This fraction of β-catenin was phosphorylated at Y654. In IAR-20 cells stimulated with EGF, IAR-6-1 cells, and IAR-6-1-DNE cells, α-catenin and pY654-β-catenin co-immunoprecipitated with IAC proteins vinculin, α-actinin, and zyxin. It was established that various RTKs including EGFR can co-localize with IACs, interact with components of IACs [53,54,55], and be activated by integrin-mediated adhesion [56,57]. Proteomic analysis demonstrated that a large number of kinases recruited to IACs phosphorylate proteins associated with adhesion complexes [58]. We propose that weakening of cell–cell adhesion in cells undergoing EMT causes redistribution of the E-cadherin/β-catenin/α-catenin complexes; the complexes that are not required for the formation of AJs can accumulate at IACs through the interaction of α-catenin with α-actinin or vinculin, which leads to the phosphorylation of β-catenin at Y654 and dissociation of E-cadherin. At IACs, pY654-β-catenin may stimulate cell migration through the formation of a complex with the multi-functional protein APC, whose role in microtubule-dependent actin-driven membrane protrusion has been described in several extensive reviews [59,60]. Recently, it was shown that APC regulates actin assembly at IACs and microtubule- and actin-dependent IAC turnover, which are critical for directional cell migration [61,62]. Partial co-localization of β-catenin and APC clusters was observed at the ends of cell protrusions [63,64], but the role of interactions between β-catenin and APC in cell migration requires further study.

Thus, we showed that in cells undergoing dynamic EMT and in neoplastically transformed epithelial cells in hybrid and fully mesenchymal states, cell–cell adhesion proteins not participating in the formation of AJs together with components of IACs may be involved in the stimulation of cell migration.

## Data Availability

Data is contained within the article or Supplementary Material.

## Supplementary Materials

The following supporting information can be downloaded at: https://www.mdpi.com/article/10.3390/cells13090780/s1: Figure S1. α-Catenin forms complexes with E-cadherin and β-catenin in IAR-6-1 and IAR-6-1-DNE cells. Figure S2. An IAR-6-1 cell transiently transfected with F-tractin-tdTomato in contact with other cells. Figure S3. Depletion of β-catenin leads to loss of staining for pY654-β-catenin. Video S1. EGF-induced EMT in IAR-20 cells. Video S2. EGF-induced EMT in IAR-20 cells stably expressing F-tractin-tdTomato. Video S3. Migration of IAR-6-1 cells. Video S4. Migration of IAR-6-1-DNE cells. Video S5. An IAR-6-1 cell transiently transfected with F-tractin-tdTomato in contact with other cells. Video S6. Migration of a single IAR-6-1-DNE cell transiently transfected with F-tractin-tdTomato. Video S7. MDA-MB-468 cells treated with EGF. Video S8. EGF-induced migration in MDA-MB-468 cells expressing exogenous α-catenin.
