# Supplementary material for: Actin Cytoskeleton Remodeling Accompanied by Redistribution of Adhesion Proteins Drives Migration of Cells in Different EMT States
Source: Cells. 2024 May 2;13(9):780. doi: 10.3390/cells13090780 (PMC11083118; doi:10.3390/cells13090780)
Supplement: Supplementary file 1 [file cells-13-00780-s001.zip › Supplementary information.pdf]

Supplementary information

Actin cytoskeleton remodeling accompanied by redistribution of adhesion proteins drives migration of cells in different EMT states

Alla S. Ilnitskaya [1,†], Nikita I. Litovka [1,†], Svetlana N. Rubtsova [1], Irina Y. Zhitnyak [1,2] and Natalya A. Gloushankova [1,*]

[1] Institute of Carcinogenesis, N.N. Blokhin National Medical Research Center of Oncology, 24 Kashirskoye Shosse, 115478 Moscow, Russia
[2] Department of Molecular Genetics, University of Toronto, 661 University Ave, MaRS West, Toronto, ON 5MG 1M1, Canada
* Correspondence: n.gloushankova@ronc.ru
† These authors contributed equally to this work.

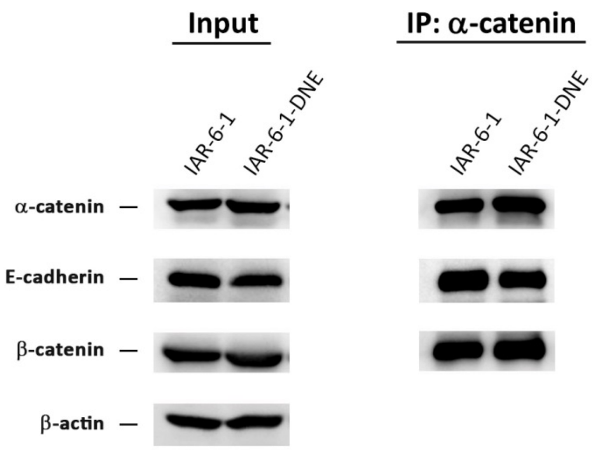

Supplementary figure S1. α-Catenin forms complexes with other adhesion proteins in IAR-6-1 and IAR-6-1-DNE cells. Total cell lysates of sparse cultures of IAR-6-1 and IAR-6-1-DNE were immunoprecipitated for α-catenin and probed with the indicated antibodies.

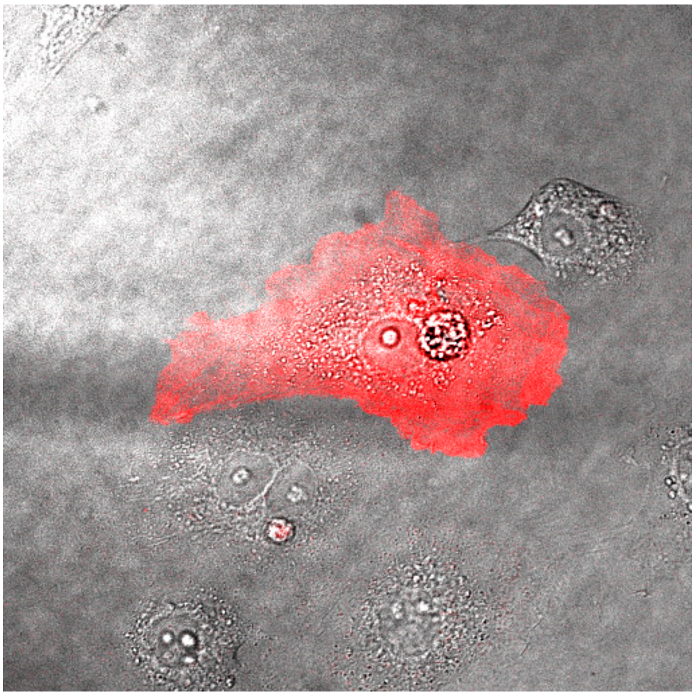

Supplementary figure S2. An IAR-6-1 cell transiently transfected with F-tractin-tdTomato in contact with other cells. Frame #1 from Supplementary Movie 5. Superimposition of DIC and red channels.

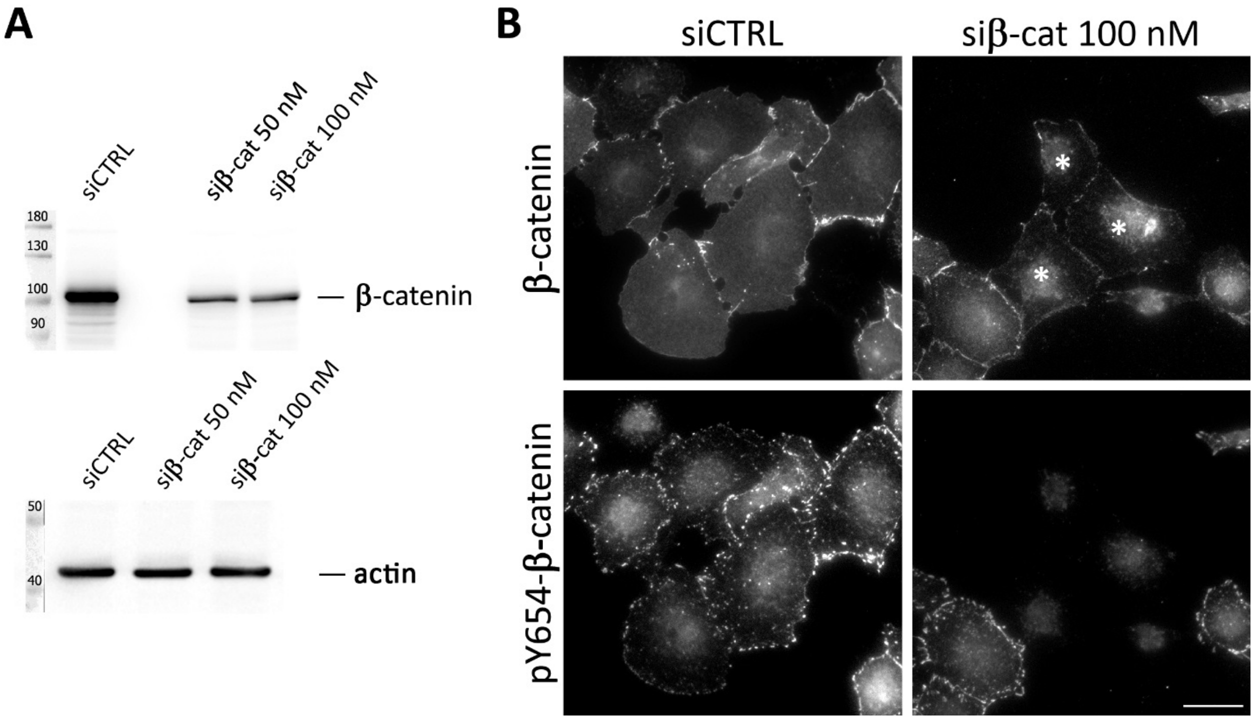

Supplementary figure S3. Depletion of β-catenin leads to loss of staining for pY654-β-catenin. (**a**) Western blot for β-catenin in IAR-20 cells treated with EGF for 20 min 48 h after transfection with either control siRNA or siRNA to β-catenin. (**b**) Immunofluorescent staining for β-catenin and pY654-β-catenin. All cells treated with control siRNA stain for pY654-β-catenin on the free edges. Cells transfected with siRNA to β-catenin (indicated by asterisks) no longer stain for pY654-β-catenin. Scale 20 μm.

Legends to Supplementary Videos

Supplementary Video S1. EGF-induced EMT in IAR-20 cells. 1 frame/5min.

Supplementary Video S2. EGF-induced EMT in IAR-20 cells stably expressing F-tractin-tdTomato. 1 frame/min

Supplementary Video S3. Migration of IAR-6-1 cells. 1 frame/5min. Arrowheads denote newly formed AJs. Arrowheads denote newly formed cell-cell contacts

Supplementary Video S4. Migration of IAR-6-1-DNE cells. 1 frame/5min

Supplementary Video S5. An IAR-6-1 cell transiently transfected with F-tractin-tdTomato in contact with other cells. 1 frame/min

Supplementary Video S6. Migration of a single IAR-6-1-DNE cell transiently transfected with F-tractin-tdTomato. 1 frame/min

Supplementary Video S7. MDA-MB-468 cells treated with EGF. 1 frame/10 min

Supplementary Video S8. EGF-induced migration in MDA-MB-468 cells expressing exogenous α-catenin. 1 frame/10 min
